# Comparison between Thai and Indian Adolescents’ Self-Figure Drawing as Child Abuse Art-Based Assessment

**DOI:** 10.3390/children11091101

**Published:** 2024-09-08

**Authors:** Nisara Jaroenkajornkij, Meghna Girish, Bussakorn Binson, Rachel Lev-Wiesel

**Affiliations:** 1 MA Expressive Arts Therapy Programme, Chulalongkorn University, Bangkok 10330, Thailand; bussakorn.s@chula.ac.th; 2 The FAA–Emili Sagol Creative Arts Research and Innovation for Well-Being Center, Chulalongkorn University, Bangkok 10330, Thailand; rlev@univ.haifa.ac.il; 3 School of Creative Arts Therapies, University of Haifa, Haifa 3498838, Israel; meghsgkg@gmail.com; 4 The Emili Sagol Research Center for Creative Arts Therapies, University of Haifa, Haifa 3498838, Israel; 5 Research Center for Innovation in Social Work, Tel-Hai College, Qiryat Shemona 1220800, Israel

**Keywords:** self-figure drawing, child abuse detection, cultural context, India, Thailand

## Abstract

Background/Objectives: The current study compared Self-Figure drawings from Thai and Indian adolescents to assess the cross-cultural applicability of a child abuse assessment tool. The research aims to understand the extent to which distinctions or similarities arise in Self-Figure drawings among adolescents from two culturally similar yet distinct backgrounds characterized by differences in religious affiliations, socioeconomic contexts, and political environments. Methods: Employing a mixed-methods approach, the study utilized quantitative measures, including the Traumatic Events Checklist (TEQ-5) and Medical Somatic Dissociation Questionnaire (MSDQ), alongside a qualitative analysis of Self-Figure drawings. Ethical approval was obtained with waived informed consent, and a convenience sample of 386 adolescents aged 13–18 years (193 from Thailand [M = 14.8, SD = 1.73; 135 females (69.9%) and 58 males (30.1%)], and 193 from India [M = 15.2, SD = 1.64; 135 females (69.9%) and 58 males (30.1%)]), who reported experiencing child abuse, participated in the study by completing questionnaires and drawing themselves. Results: The analysis revealed that Thai adolescents had higher MSDQ scores, while Indian adolescents exhibited more prominent pictorial indicators. Indian participants reported experiencing a broader range of event types, whereas Thai adolescents predominantly depicted verbal or emotional abuse. Variations in pictorial indicators were found significant, except for hair, cheek or chin, omitted legs or feet, and omitted eyes. Conclusions: These findings contribute to the understanding of how cultural factors influence adolescents’ self-representations through drawing. The differences in pictorial indicators highlight the nuanced variations within similar cultures, emphasizing the cultural specificity of self-expression.

## 1. Introduction

Child abuse is a pervasive public health issue that leads to detrimental physical and psychological health effects for children under 18 years of age, both in the short term and long term. Child abuse, including childhood sexual abuse (CSA), childhood physical abuse (CPA), and childhood emotional abuse (CEA), affects approximately 12.7%, 22.6%, and 6.3% of children globally, respectively, with rates influenced by various cultural, economic, and social factors [1,2,3]. Despite its prevalence, with around one million children impacted annually [4], there remains a significant gap between the incidence of abuse and disclosure rates, further complicated by a lack of effective assessment tools [5,6]. Child abuse often leads to delayed disclosure, complicated by personal, social, and cultural factors.

Despite these challenges, disclosure is essential as it initiates the process of stopping the abuse and securing emotional and legal support. Prolonged delays, particularly into adulthood, aggravate the negative effects of abuse, such as trauma, cognitive delays, and an increased risk of re-victimization [7,8,9,10]. Disclosure is a dynamic process influenced by factors like self-blame, shame, fear, and a lack of appropriate vocabulary, all of which contribute to non-disclosure or delays in seeking help [11,12,13]. Failure to detect abuse risks subjecting the child to further harm, while adult survivors may receive inadequate treatment [14]. Common assessments, including anogenital examinations, psychosocial tests, interviews, and victim disclosure, have limitations, such as difficulties in confirming transient physical signs [15] and challenges related to the child’s reluctance to disclose or narrative incoherence in their testimonies [16]. There is a pressing need for non-intrusive, projective, and child-friendly abuse detection tools.

Drawing has long been regarded as a form of human expression, as it reveals thoughts, attitudes, conflicts, and personality traits that affect emotional well-being [17]. This has prompted scientific research into its potential as a research tool and method of psychological assessment [18,19]. A version of the Draw-A-Person test [20], known as the Self-Figure drawing, prompts individuals to “Draw yourself.” It is recognized as a valuable psychological assessment tool for screening child abuse, as developed by Lev-Wiesel [21]. Recent research has highlighted the efficacy of Self-Figure drawings in distinguishing three forms of child abuse—emotional, physical, and sexual—in countries like Israel, Thailand, and India. These studies employed indicators within the drawings to detect abuse [22,23,24]. Adolescents’ self-portraits can offer insights into nonverbal and culturally based developmental differences in their self-perceptions [25,26].

However, research exploring the cultural nuances depicted in Self-Figure drawings has been lacking. There are a small number of studies on the comparison between cultures on the Self-Figure drawing. A comparison between Thais’ and Israelis’ Self-Figure drawings found differences in body size and shape and the addition of religious and cultural symbols [27]. A study examining how individuals express themselves in drawings found that Euro-Canadians depicted a larger “self” compared to Chinese participants. The size of the “self” was also larger in scenarios of success than in scenarios of failure. Furthermore, Euro-Canadians were significantly more likely than Chinese participants to include a face on their “self” [28]. Cultural and social influences between U.S. and Japanese children are greatly reflected in their drawings. Compared to the U.S., Japanese children often used exaggerated perspectives, bird’s-eye views, and multi-perspective views [29].

The largest religion in Thailand is Buddhism, while in India it is Hinduism. The similarities between Buddhism and Hinduism are evident in their concepts, symbols, and practices. For instance, both religions share similar terminologies for religious concepts like samsara (the endless cycle of rebirth), karma (cause and effect), and dharma (moral order). They also use comparable symbols such as mudras (hand gestures with specific meanings) and the dharma chakra (wheel of law). Additionally, their practices overlap in the use of mantras (the melodic utterance of syllables or words) and meditation (physical, mental, and spiritual enhancement) [30]. Although there are more similarities than differences, the differences in social norms, regimes, and socioeconomic situations can influence individuals, as seen in Table 1 below [31,32,33,34,35,36,37,38,39,40,41].

These differences can be reflected in Self-Figure drawings, which offer insights into the inner world and unconscious factors. Therefore, this study aims to fill this gap and make a significant contribution to the field by investigating the differences observed in the drawings of Thai and Indian adolescents. The research seeks to understand the extent to which distinctions or similarities arise in Self-Figure drawings among adolescents from two culturally similar yet distinct backgrounds. These backgrounds are characterized by differences in religious affiliations, socioeconomic contexts, and political environments.

### 1.1. Child Abuse in Thailand and India

Child Sexual Abuse (CSA) encompasses any sexual interaction between a child and an adult or older child for gratification, including acts such as fondling, rape, voyeurism, or exposure to pornography [42]. This form of abuse often results in significant physical and mental health consequences for the victim [43]. Child Physical Abuse (CPA) involves intentionally harming or putting a child at high risk of harm through physical force, such as hitting or shaking [44,45]. This form of abuse often leads to psychological issues such as depression, anxiety, and substance misuse, as well as physical health conditions like chronic pain and asthma [46]. Child Emotional Abuse (CEA) encompasses verbal or nonverbal aggression aimed at a child’s well-being, which can include humiliation and emotional neglect [47]. The enduring effects of CEA may manifest as depression, emotion dysregulation, and interpersonal difficulties [48]. According to the Union for Civil Liberty [49], there was an increase in reported cases of child abuse, with 15,000–16,000 cases documented between 2019 and 2021. Among these cases, 40% involved children under the age of 20, and 47% of the victims did not know where to seek help. Thailand has approximately 42 million children, with an estimated 100 children being abused every hour. Online sexual exploitation and abuse is a critical issue in Thailand, with approximately 9% of internet users aged 12–17 being victims of such crimes, including coercion into sexual activities or unconsented sharing of their sexual images [50]. Prevalence rates for India’s CSA, CPA, and CEA range from around 40%, 75%, and 84%, respectively [51,52]. According to UNICEF 2012, there are more than 25 million orphans or abandoned children and about 44 million destitute children in India. Thai and Indian cultures emphasize obedience, conformity, respect for elders, and social interdependence [53,54]. Indian adolescents may perceive parental control as more acceptable and normal due to the prevalent use of authoritarian parenting styles, where parents prioritize obedience and foster interdependent behavior in their children [55]. In India, authoritative parenting styles are associated with positive outcomes such as increased self-efficacy, an internal locus of control, and notable academic achievements, despite the evolving expectations of the current generation, which often seeks greater autonomy [56]. Similar to Indian culture, the authoritative parenting style has been found to be predominant among most Thai parents. However, Thai parents prioritize obedience and attentiveness while also leaning towards compromise and conflict avoidance. As a result, their parenting approach falls somewhere between authoritative and permissive, rather than strictly aligning with an authoritarian approach [57].

### 1.2. Thai and Indian Cultures

Thailand and India, both collectivistic nations, prioritize group needs and goals alongside robust family structures [58]. In Thai culture, corporal punishment remains prevalent as a disciplinary measure, often viewed as an expression of parental love [59]. Similarly, in Indian culture, disciplining children frequently involves methods like pinching or twisting their ears [52]. Both cultures emphasize hierarchy and strong familial bonds, with expectations for children to obey parental authority and fulfill their wishes [60]. Families from middle to high socioeconomic backgrounds in both countries prioritize their children’s future success by enrolling them in private tutorial institutes and encouraging participation in extracurricular activities [61]. The preservation of virginity until marriage is culturally significant in both Thai and Indian societies, carrying implications for family reputation and heritage [62,63]. Differences and similarities are summarized in Table 2 below.

### 1.3. Self-Figure Drawing for Child Abuse Detection

Self-Figure drawing serves as an assessment tool for specific psychological traits such as self-injury tendencies [64], learning challenges [65], and dissociative disorders [66]. Studies have demonstrated its efficacy in evaluating children’s mental health, with features like “clawed fingers,” “visible teeth,” and “exaggerated shoulder width” potentially indicating aggression [65]. Lev-Wiesel [21] utilized the DAP test to identify common indicators in drawings of adults sexually abused during childhood, revealing features like facial lines (double, hollow, or shaded), eyes (dots, omission, shading, hollowness), hands and arms (cut off, clinging, detached, omitted), and genitals (shaded, blocked off). These indicators were supported in studies examining dissociative identity disorder [66] and self-mutilation behaviors among sexually abused adolescents [64]. McIness [67] observed that drawings of sexually abused children often depict them as small, incomplete, lacking facial features, missing limbs, and appearing suspended. A study by Popa-Velea et al. [68] suggested that the DAP test could be a valuable component of the diagnostic toolkit for child physical abuse (CPA), highlighting indicators such as small drawing dimensions, aggressive markers (e.g., strongly shaded hair, peculiar noses, presence of teeth), dominant, sharp lines, and large hands.

With this background, our research aims to address the following questions: (1) What are the differences and similarities in traumatic events experienced by Thai and Indian adolescents? (2) How do the Self-Figure drawings of Thai and Indian adolescents compare in terms of similarities and differences? (3) To what extent do MSDQ scores and Self-Figure drawings correlate among adolescents from Thai and Indian cultures?

## 2. Materials and Methods

### 2.1. Participants and Procedure

A convenience sample of 386 adolescents, including 135 females and 58 males from both Thai and Indian populations, participated in this study, conducted between 11 February and 30 September 2021. The participants were selected through public lectures on mental health and Self-Figure drawing offered in schools and communities in Thailand and India. Following these lectures, authorities granted permission to collect data from adolescents who met the inclusion criteria: aged between 13 and 18 years, and either receiving treatment for mistreatment by welfare services or self-reporting experiences of abuse through the questionnaire. Exclusion criteria included adolescents outside the specified age range or those without a history or report of mistreatment. The mean age of the Thai adolescents was 14.8 years (SD = 1.73), while the mean age of the Indian adolescents was 15.2 years (SD = 1.64).

Ethical standards were rigorously adhered to throughout the study. Ethical approval was obtained from the relevant ethics committees in both Thailand and India. Informed consent was waived by the ethics committees due to concerns that abusive guardians might prevent their children from participating if required to provide consent. Instead, authorities provided consent on behalf of the adolescents. Participants were fully informed about the study’s nature and assured of their anonymity, confidentiality, and voluntary participation. Personal data were anonymized, with all identifying information removed to protect participant privacy. Additionally, participants were offered access to counseling or therapy services if they experienced any distress during or after the study. The measures were administered in Thai for the Thai participants and in Hindi and Malayalam for the Indian participants to ensure full comprehension.

#### 2.1.1. Demographic Sheet

The participants completed the demographic sheet, including details such as age and gender, along with a life events checklist. This checklist queried whether they had experienced specific events such as a car accident, physical assault, terror attack or war, hospitalization due to illness, sexual abuse, loss of family member, social exclusion, and other events.

#### 2.1.2. Medical Somatic Dissociation Questionnaire (MSDQ)

The MSDQ [6] is a questionnaire with 30 items eliciting the severity of the following symptoms: dissociation, depression, and somatization. Each item is scored on a 5-point Likert scale from 0 (nothing) to 4 (extremely). The Cronbach’s alpha for internal consistency of the questionnaire is 0.93 [6]. It is also seen that the questionnaire has strong convergent validity and known-group validity [6]. For the current study, Cronbach’s alpha for MSDQ was 0.88 and 0.87 for the Thai and Indian samples, respectively.

#### 2.1.3. Self-Figure Drawing

The Self-Figure drawing [67] is a version of the Draw-A-Person test developed by Machover [20]. During the portrait orientation, children were instructed to draw themselves on an A4 paper sheet. When asked about how to draw the figure, they were informed that they were free to interpret the instructions however they wished. Drawing skill was irrelevant in this test since the analysis focused on specific indicators of the drawings. For each indicator of abuse, the art therapists rated the level of obviousness, with 1 being not at all obvious and 4 being very obvious. Signs of sexual abuse in the drawings are eyes (emphasized, shadowed, dots, or omitted), arms and hands (emphasized, big, shadowed, or omitted), cheek/chin (double or emphasized), and genital area (disconnected upper body from lower, shadowed, or omitted) [21,66,67]. Signs of physical abuse in the drawings are nose (emphasized, big, or shadowed), forehead (emphasized, or shadowed), eyebrows (emphasized, or shadowed), ears (double, emphasized, or shadowed), and standing hair [69]. Signs of emotional abuse in the drawings are slanted figure, tiny head, omission of hands, omission of legs and feet, monster and grotesque figure, disintegrated body, arm and leg asymmetry, omission of the neck, tiny figure, omission of eyes, shading of the face, and feet being pressed together [70]. Indicators and ratings can be seen in Table 3 below.

#### 2.1.4. Data Analysis

Continuous variables were presented using means and standard deviations, while categorical variables were depicted using frequencies and percentages. Only indicators with good reliability from the ratings were utilized for analysis. A chi-square or Fisher’s exact test was conducted to compare categorical variables between Thais and Indians. Given that the variables did not follow a normal distribution, the Mann–Whitney U Test was employed to compare continuous variables between the two groups. To evaluate the associations between the index for each abuse type and MSDQ scores, the Spearman correlation coefficient was utilized.

## 3. Results

The study revealed significant differences between Thai and Indian adolescents in terms of traumatic experiences, psychological symptoms, and pictorial indicators of abuse. These findings are further stratified by gender to explore potential gender-specific differences within each cultural context.

As shown in Table 4, the average age of participants was slightly higher in the Indian group (M = 15.2, SD = 1.64) compared to the Thai group (M = 14.8, SD = 1.73). A higher percentage of Indian adolescents reported experiencing certain traumatic events, such as hospitalization due to illness (22.3% in India vs. 3.1% in Thailand) and the loss of a family member (59.1% in India vs. 2.6% in Thailand). Conversely, verbal or emotional abuse was reported almost exclusively by Thai participants (98.4% in Thailand vs. 0% in India).

As indicated in Table 5, Indian adolescents generally scored higher on pictorial indicators of abuse across various forms of abuse compared to their Thai counterparts, with notable differences when stratified by gender. Indian males had the highest Physical Abuse (PA) Index (M = 9.7, SD = 2.4), followed by Indian females (M = 9.0, SD = 2.3), while Thai males and females scored lower (M = 7.5, SD = 2.8 for males; M = 7.0, SD = 2.8 for females), with indicators like the nose and eyebrows more pronounced in Indian males. For Sexual Abuse (SA), Indian females had the highest SA Index (M = 11.0, SD = 2.6), followed by Indian males (M = 10.2, SD = 2.6), with Thai participants scoring lower (M = 8.3, SD = 2.3 for females; M = 7.7, SD = 2.3 for males), and genital area indicators more evident in Indian females. In terms of Emotional Abuse (EA), Thai females exhibited a higher EA Index (M = 12.2, SD = 2.7) compared to Thai males (M = 11.4, SD = 2.7), while Indian adolescents showed similar patterns (M = 11.4, SD = 2.4 for females; M = 10.7, SD = 2.4 for males), with slanted figures and tiny heads more common in Indian females, and omitted eyes and feet pressed together more frequently observed in Thai females.

**Figure 1:**
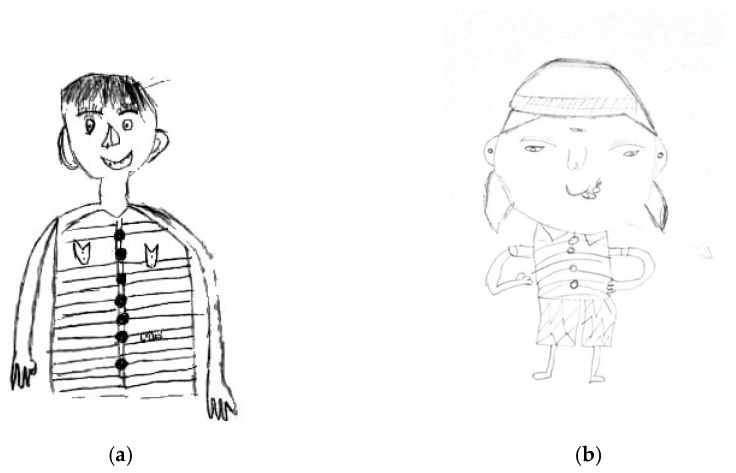
The physical abuse indicators of the same age and gender participants between a Thai male adolescent aged 13 (**a**) versus an Indian male adolescent aged 13 (**b**) nose: emphasized, big, or shadowed; forehead: emphasized or shadowed; eyebrows: emphasized or shadowed; ears: double, emphasized or shadowed, and standing hair.

Figure 1 shows the following physical abuse indicators: an emphasized, big, or shadowed nose; an emphasized or shadowed forehead; emphasized or shadowed eyebrows; doubled, emphasized, or shadowed ears; and standing hair. 

**Figure 2:**
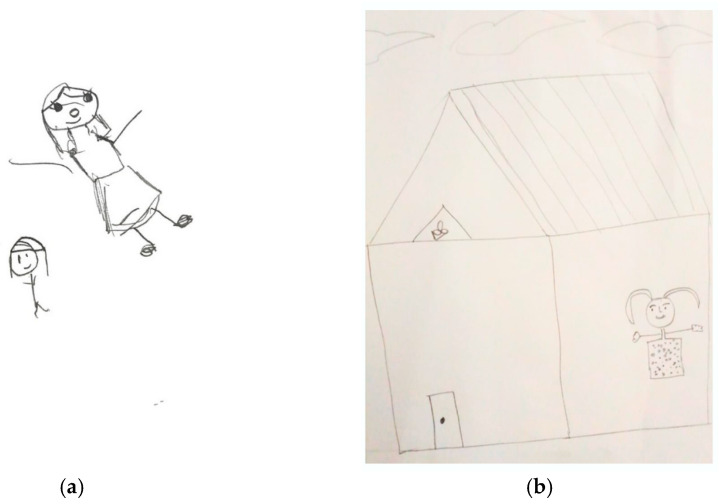
The sexual abuse indicators of the same age and gender participants between a Thai female adolescent aged 15 (**a**) versus an Indian female adolescent aged 15 (**b**) eyes: emphasized, shadowed, dots, or omitted; arms and hands: emphasized, big, shadowed, or omitted; cheek/chin: double or emphasized; and genital area: disconnected upper body from lower, shadowed, or omitted.

The following indicators of sexual abuse can be seen in Figure 2: emphasized, shadowed, dots, or omitted eyes; emphasized, big, shadowed, or omitted arms and hands; doubled or emphasized cheek/chin; and genital area: disconnected upper body from lower, shadowed, or omitted.

**Figure 3:**
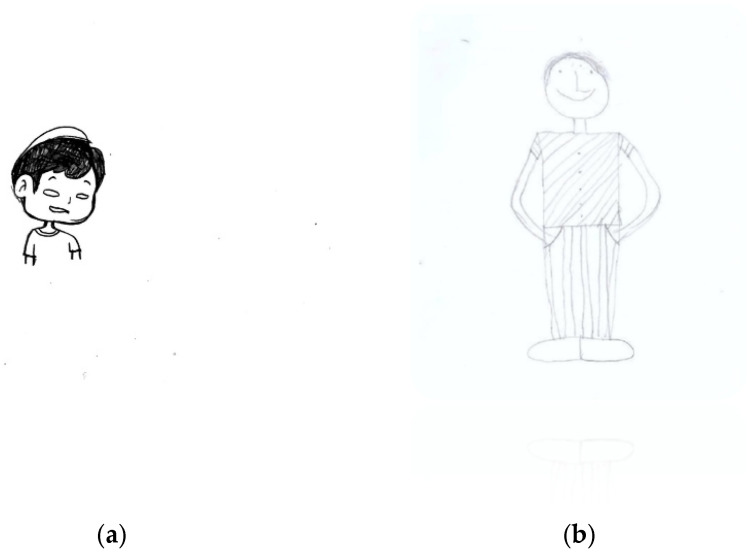
The emotional abuse indicators of the same age and gender participants between a Thai male adolescent aged 15 (**a**) versus an Indian male adolescent aged 15 (**b**) slanted figure, tiny head, omission of hands, omission of legs and feet, monster and grotesque figure, disintegrated body, arm and leg asymmetry, omission of the neck, tiny figure, omission of eyes, shading of the face, and feet being pressed together.

Figure 3 illustrates several indicators of emotional abuse, including a slanted figure, tiny head, omission of hands, omission of legs and feet, monster and grotesque figure, disintegrated body, arm and leg asymmetry, omission of the neck, tiny figure, omission of eyes, shading of the face, and feet being pressed together.

Table 6 presents a group-wise comparison of categorical variables between Thais and Indians. The table shows the number (N) and percentage (%) of individuals in each group who experienced specific events, along with the corresponding *p*-values to indicate statistical significance. Events such as physical assault, hospitalization due to illness, loss of a family member, social exclusion, verbal or emotional abuse, and other events display statistically significant differences between the two groups (*p* < 0.05). Specifically, Indians experienced a higher incidence of hospitalization, loss of family members, and no traumatic events compared to Thais. On the other hand, Thais had significantly higher rates of verbal or emotional abuse. Events like car accidents, terror attacks, sexual abuse, and others were not significantly different between the groups (NS = nonsignificant).

Table 7 shows that Thai adolescents had significantly higher scores on the Medical Somatic Dissociation Questionnaire (MSDQ) across all dimensions, indicating more severe psychological symptoms compared to Indian adolescents. When stratified by gender, Thai females exhibited the highest scores in somatization (M = 14.2, SD = 3.85), depression (M = 21.1, SD = 5.58), and dissociation (M = 20.8, SD = 5.09), compared to Thai males (Somatization: M = 12.5, SD = 4.02; Depression: M = 18.5, SD = 5.62; Dissociation: M = 18.8, SD = 5.31). Indian females and males had lower scores across all dimensions, with Indian females showing slightly higher scores than males (Somatization: M = 3.8, SD = 3.08 vs. M = 3.4, SD = 3.17; Depression: M = 6.8, SD = 5.48 vs. M = 6.3, SD = 5.42; Dissociation: M = 4.4, SD = 4.13 vs. M = 4.0, SD = 4.11).

Table 8 provides a group-wise comparison of age and MSDQ scores using the Mann–Whitney U test. The test statistics include the Mann–Whitney U, Wilcoxon W, Z-score, and corresponding *p*-values. The comparison shows that age is significantly different between the groups (*p* = 0.009). Additionally, the MSDQ subscales—somatization, depression, and dissociation—along with the total MSDQ score, all show highly significant differences between the groups, with *p*-values less than 0.001. These results suggest that the groups differ substantially in terms of both age and the levels of somatic, depressive, and dissociative symptoms, as well as overall distress.

Table 9 presents a group-wise comparison of ratings for various pictorial indicators and abuse indices (PA: Physical Abuse, SA: Sexual Abuse, EA: Emotional Abuse) between Indian and Thai participants using the Mann–Whitney U test. The table shows the number of participants (N), the mean rank, and the sum of ranks for each indicator. The results reveal that Indian participants had higher mean ranks for most indicators related to physical and sexual abuse, including nose, forehead, eyebrows, ears, arms or hands, and the genital area, indicating more frequent or severe representations in their drawings. In contrast, Thai participants had higher mean ranks for indicators such as the eyes, tiny head, and omitted hands or arms, often associated with emotional abuse. Additionally, the abuse indices (PA, SA, and EA) reflect these differences, with Indian participants generally showing higher rankings for physical and sexual abuse indicators, while Thai participants ranked higher for emotional abuse indicators. The differences in rankings between the two groups suggest variations in the ways physical, sexual, and emotional abuse are expressed pictorially in their respective drawings.

Table 10 presents the test statistics from the Mann–Whitney U test used to compare the ratings of various pictorial indicators and abuse indices between Indian and Thai participants. The test statistics include the Mann–Whitney U, Wilcoxon W, Z-scores, and the corresponding *p*-values for each variable. Significant differences (*p* < 0.001) were found for several indicators, including the nose, forehead, eyebrows, ears, physical abuse (PA) index, eyes, arms or hands, genital area, sexual abuse (SA) index, slanted figure, tiny head, omitted hands or arms, feet pressed, and leg or arm asymmetry. These findings suggest that these pictorial elements and abuse indices were rated differently between the two groups, indicating distinct representations of abuse. Notably, the genital area had the most substantial difference, with a Z-score of −13.128. However, for some indicators like hair, cheek or chin, and omitted legs or feet, the differences were not statistically significant (NS), indicating similarity between the groups in how these features were depicted. The emotional abuse (EA) index also shows a significant difference between the groups (*p* = 0.013), though the effect is smaller compared to physical and sexual abuse indicators. This suggests clear variations in how abuse is visually represented by the two nationalities across multiple forms of abuse, with physical and sexual abuse showing particularly strong differences.

Table 11, Table 12 and Table 13 highlight significant correlations between age, MSDQ scores, and pictorial indicators of abuse stratified by gender. For instance, older age was positively correlated with the CPA Index and certain pictorial indicators like the forehead and eyebrows across both genders, indicating that as adolescents age, the indicators of physical abuse in their drawings become more pronounced. Gender differences in correlations suggest that psychological symptoms and their manifestation in drawings may vary between males and females within each cultural context. The analysis showed that Indian females who reported CSA had a notably higher Sexual Abuse (SA) Index, with exaggerated arms and hands and prominent genital area indicators. Thai participants affected by CSA also demonstrated distinct patterns, such as emphasizing eyes and omitting body parts.

## 4. Discussion

The study analyzed pictorial indicators of sexual, physical, and emotional abuse in Self-Figure drawings of abused adolescents from Thailand and India, alongside MSDQ scores for the entire sample. Thai adolescents exhibited higher MSDQ scores, whereas Indian adolescents showed higher scores for pictorial indicators. Group comparisons indicated differences in all events except car accidents, terror attacks or war, and sexual abuse. Indian adolescents reported experiencing a broader range of event types, while verbal or emotional abuse was predominant among Thai adolescents. Some pictorial indicators, such as hair, cheek or chin, omitted legs or feet, and omitted eyes, did not show significant differences between the two groups. MSDQ scores correlated significantly with abuse indexes derived from pictorial indicators. However, certain indicators, such as ears and hair from physical abuse and eyes and cheek or chin from sexual abuse, showed no significant correlation. Arm or leg asymmetry, tiny figure, and shading of the face from emotional abuse were found to significantly correlate with depression, somatization, and dissociation, respectively.

### 4.1. Differences in Self-Report Questionnaires

The ambiguity and confusion among Indian adolescents regarding whether an event they experienced was abusive, especially emotional abuse, can be seen from the discrepancies in events marked in the TEQ. Unlike the Thai sample, the entire Indian sample omitted marking the category of “Verbal or emotional abuse”, indicating a notable difference in perception between the two groups. Despite being assured of anonymity, individuals are reluctant to admit their experiences on a checklist and the MSDQ. This reluctance is consistent with findings from a previous study conducted among Indian adolescents [71]. Considering the societal context of India, characterized by high levels of poverty and child neglect [72,73] leading to physical issues and elevated mortality rates [74], Indian adolescents may find it easier to admit certain events, such as hospitalization due to illness or loss of family members, even marking “no event” or “other event” in the TEQ. From a young age, Indian children are trained and expected to be resilient and capable of enduring the challenges they face [75]. This cultural expectation could explain why Indian adolescents may find it easier to admit certain events over others and subsequently score lower in the MSDQ compared to their Thai counterparts. Despite assured anonymity, many Indian participants reported no experience of abuse, indicating a reluctance to disclose, even anonymously. This pattern aligns with previous findings where Indian children expressed concerns about expressing themselves, reflecting a broader reluctance among Indians to disclose instances of abuse [76].

As Thai culture places a high value on harmony, conformity, and relationships [77], Thai individuals often prefer a non-confrontational approach in order to prevent conflicts and the associated discomfort [78]. The anonymity offered by the questionnaire may help alleviate the reluctance to disclose traumatic experiences and provide an outlet to express repressed feelings [79], potentially explaining why the Thai sample scored higher on the MSDQ but lower on the pictorial indicators. Drawings, being non-intrusive and non-threatening, showed higher scores for Indian adolescents in the pictorial indicators.

### 4.2. Differences in Pictorial Indicators in Self-Figure Drawing

In terms of indicators of CPA, Indian adolescents scored higher than Thai adolescents in areas such as the nose, forehead, eyebrow, and ears. Particularly noteworthy is the prominence of the nose, often considered a phallic symbol [80], which emerged significantly more prominently among Indian adolescents compared to Thais. This discrepancy may be attributed to the higher prevalence of physical abuse and the symbolic significance of the nose within Hindu religious narratives, such as stories where a female character’s nose is slashed by a male, implying dominance and control [81]. Thai adolescents frequently portrayed themselves with small or simplistic noses, likely aiming to convey an image of non-threatening behavior. This stands in contrast to an exaggerated or emphasized nose, which often signifies aggression and a tendency towards action [69]. The significance of the forehead in instances of physical abuse might be attributed to the association between various forms of physical abuse, including blunt force trauma or abusive head injuries [51], which are more prevalent among Indian adolescents [82]. The shape of eyebrows plays a crucial role in swiftly identifying threatening facial expressions [83]. Recent research suggests that Indian adolescents are adept at discerning facial cues [84]. Conversely, in Thai culture, facial expressions tend to emphasize the mouth. As a result, Thai adolescents often depict themselves with wide, smiling mouths, symbolizing happiness and contentment. This aligns with findings by Grothaus [85], indicating that Thai adolescents utilize smiles or laughter as coping mechanisms to navigate challenges and evade conflicts. In Indian culture, pinching or twisting a child’s ears is a prevalent disciplinary method used by parents, guardians, and teachers to address misbehavior [52]. Conversely, spanking, whether administered by hand or using items like wooden sticks, clothes hangers, or feather dusters, is more common in rural areas of Thailand [86]. This variation in disciplinary practices may account for the higher prevalence of this particular indicator in Indian samples compared to Thai samples.

Among indicators of CSA, Indian adolescents exhibited higher scores for arms or hands and genital area compared to their Thai counterparts. However, Thai adolescents scored higher on the eye indicator, indicating feelings of concealment, suspicion of others, helplessness, anxiety, and a fear of being seen [69]. This observation may reflect a cultural inclination toward secrecy, conflict avoidance, and the preservation of reputation, which are highly valued in Thai culture. The emphasis on the genital area among Indian adolescents may be attributed to cultural beliefs that uphold the sanctity of female virginity, which is expected to be preserved until marriage. Additionally, in Indian culture, sexual matters are often regarded as sensitive topics that are kept concealed due to concerns related to family honor. This can lead to feelings of silence, embarrassment, shame, and blame surrounding discussions about sexuality [63]. Child sexual abuse frequently involves the use of arms and hands, particularly in actions such as the rubbing or massaging of private parts [87]. This tendency is heightened in the Indian context, where perpetrators may encourage children to touch or fondle their genitals [88]. Therefore, it is likely that arms or hands stood out more in the drawings of Indian adolescents due to these cultural and contextual factors.

For indicators of CEA, Indian adolescents scored higher compared to their Thai counterparts, particularly in terms of slanted figures and arm or leg asymmetry. A slanted figure may suggest an unstable ground or an inability to stand securely, while arm or leg asymmetry could indicate impulsivity [70]. Moreover, asymmetric arm positions may also signify a cry for help, low self-esteem, and a tendency to avoid contact with the environment [89].

### 4.3. Similarities in Pictorial Indicators in Self-Figure Drawing

The presence of standing hair, often symbolic of anxiety and an indicator of physical abuse [24], did not show significant differences between Indian and Thai adolescents. This might be attributed to both cultures’ normalization of physical abuse and punishment [52,86]. The indicator of cheek or chin represents feelings of disgust and a desire to “vomit out” the experience of sexual abuse, associated with feelings of shame, guilt, and fear [49]. Globally, sexual abuse is perceived with disgust as an inherent emotion [90,91], which may explain why the cheek/chin indicator did not show significant differences in the group comparison. In Thai culture, feet are regarded as the lowest and dirtiest part of the body [27]. Consequently, it is customary to keep one’s feet on the floor and refrain from using them to point or push objects around [92]. Similar to Indian culture, pointing one’s feet towards an altar, teacher, or elder is considered disrespectful, while touching the feet of an elder or visiting religious shrines barefoot is seen as sacred [93,94]. This shared cultural perspective may explain why the indicator of omitted feet or legs is observed as a sign of emotional abuse in both cultures. The eyes, often considered the “window to the soul”, serve as another indicator of emotional abuse when omitted. In both collectivistic cultures, they symbolize and play a similar role in human social interaction [95]. Thai individuals frequently employ silence as a face-saving politeness strategy, deliberately avoiding or ending conversations during social interactions to prevent potential conflicts and avoid expressing inner feelings or thoughts [78]. The consistency in these indicators across cultures suggests that professionals can effectively utilize the Self-Figure drawing tool cross-culturally.

### 4.4. Correlation between MSDQ and Self-Figure Drawing for Total Sample

The MSDQ and the indexes for each form of abuse demonstrated a significant positive correlation, consistent with prior research [22,23,24]. However, ears and hair from CPA indicators did not correlate with the MSDQ scores. This may be because the sample comprised adolescents, who often experience anxiety and a strong desire for adults to listen to them [96,97]. Surprisingly, the eyes and cheek or chin indicators from CSA did not correlate with the MSDQ scores, possibly due to the disparity in scores between Thais and Indian adolescents. A tiny figure symbolizes lower self-esteem [98], correlating with the somatization subscale due to the negative correlation found between somatization and self-esteem [99]. The negative correlation between arm or leg asymmetry and impulsivity, low self-esteem, and depression [100,101] could explain the findings as arm or leg asymmetry symbolizes impulsivity, a cry for help, low self-esteem, and a tendency to avoid contact with the environment. Machover [20] found that shading on the face in human figure drawings indicates anxiety, a conclusion supported by another study showing that facial shading might reflect an individual’s self-consciousness regarding their complexion [102].

## 5. Limitations

This study has several limitations. Firstly, the unequal distribution of participants across different forms of abuse may have skewed the results. Secondly, the reliance on convenience sampling restricts the generalizability of the findings. Future research should consider alternative sampling methods and include participants from more diverse socioeconomic backgrounds. Including a non-victimized sample would also enhance the depth of the results. Despite the anonymity offered, some participants were hesitant to disclose traumatic experiences, likely due to feelings of shame, guilt, and a sense of responsibility for the maltreatment they endured, in addition to fear, desensitization, or potential threats from perpetrators. These emotional barriers are significant obstacles in accurately capturing the extent of their experiences. Future studies should consider methods to mitigate these barriers, such as creating a more supportive and trusting environment during data collection.

The recommendation to supplement self-report measures with brain imaging techniques aims to provide a more comprehensive understanding of the psychological impact of abuse. Brain imaging, such as functional MRI or PET scans, could be used to identify changes in brain regions associated with trauma, such as the amygdala or prefrontal cortex, offering objective data that complements self-reported experiences. This approach is particularly valuable in understanding the neurological underpinnings of trauma and could help validate the psychological symptoms reported by participants.

While child drawing is a widely used tool in assessing the psychological dimensions of abuse, it is not without its limitations. Criticisms include the subjective interpretation of drawings and potential cultural bias. However, despite these concerns, child drawing remains a valuable tool when integrated into a comprehensive medico-legal assessment protocol. It provides unique insights into a child’s emotional state and psychological well-being, particularly when other forms of evidence are limited or unavailable. In practice, drawings can effectively support expert opinions and help to emotionally prepare children for protected interviews, offering a non-threatening means of expression that complements more traditional methods of investigation.

Finally, the measures and methods used in this study, while robust, have their limitations. For instance, the MSDQ, while reliable, may not fully capture the cultural nuances influencing how trauma symptoms are expressed in different populations. The subjective nature of interpreting Self-Figure drawings also introduces variability that could affect the consistency of the results. These factors should be considered when interpreting the findings and in the design of future studies.

## 6. Practical Implications

The study highlights the potential of Self-Figure drawing as a valuable tool for identifying various forms of child abuse, including sexual, physical, and emotional abuse, across different cultural contexts. This method allows individuals to express traumatic experiences that might otherwise go unspoken, enabling professionals to detect early signs of abuse through specific drawing indicators.

Incorporating Self-Figure drawing into routine assessments in schools and therapeutic environments offers a non-intrusive way to identify at-risk children. Educators, counselors, and other professionals can use this tool to recognize visual cues linked to Child Sexual Abuse (CSA) and intervene promptly.

By identifying specific patterns in drawings, such as exaggerated or omitted body parts, professionals can detect CSA early, even before children can verbalize their experiences. This proactive approach aids in preventing further abuse and facilitates timely intervention, contributing to the overall well-being and protection of vulnerable children.

## Figures and Tables

**Table 1:** The differences in social norms, regimes, and socioeconomic situations.

Aspect	Thailand	India
Average monthly salary	29,502 THB (800.97 USD)	31,900 INR (383.68 USD)
Monthly cost of living	24,362 THB (661.42 USD)	28,806.1 INR (346.47 USD)
Fertility rate	1.452 births per woman	2.122 births per woman
Average year of schooling	9	7
Health care index	77.17	65.36
Life expectancy	77.92	70.62
Gini coefficient (economic inequality)	0.35 (adequate)	0.402 (high)
Crime rate	37.64 (low)	46.34 (moderate)

**Table 2:** Differences and similarities of Thai and Indian cultures.

Aspect	Thai Culture	Indian Culture
Religion	Predominantly Buddhism, with influences of Hinduism and Animism	Predominantly Hinduism, Islam, Sikhism, etc.
Social structure	Emphasis on respect for elders and maintaining harmonious relationships; family is central to social life	Strong emphasis on family values and hierarchy; joint families are common
Etiquette	Politeness and avoiding confrontation are valued; the wai (a slight bow with pressed palms) is a common greeting	Respect for elders is crucial; touching feet is a sign of respect; eating with hands is common
Child rearing practices	Strong emphasis on respect for elders; children are taught to be obedient and respectful towards parents and authority figures. Parents typically have a close relationship with their children	Emphasis on respect for authority and elders; children are often raised within extended family structures. Parents often have a strong influence on career choices and marriage decisions
Child abuse perspectives	Recognized as a problem, efforts to combat it are ongoing, but challenges remain in rural areas and among marginalized communities. The social stigma associated with child abuse but increasing efforts to address and prevent it; corporal punishment may also be used in certain contexts	Awareness is increasing, but still, issues of child labor, neglect, and abuse persist. Some challenges in reporting and addressing corporal punishment may be more common in some areas.
Power differentials	Respect for authority and seniority; societal hierarchy is evident in interpersonal interactions	Hierarchical structure is prevalent, with deference to elders and authority figures
Emotional expression	Emphasis on maintaining harmony and saving face; emotions may be expressed more subtly, with restraint in public settings. Direct expression of strong emotions, especially negative ones, may be avoided in favor of indirect communication	Expression of emotions varies by context and individual; stoicism may be valued, particularly in public settings; overt emotional displays may be seen as a sign of weakness

**Table 3:** Indicators and ratings of Self-Figure Drawing.

Indicators	4 Very Obvious	3 Obvious	2 Rather Obvious	1 Not at All
Sexual abuse				
Eyes (emphasized, shadowed, dots, or omitted)	The indicators are either clearly presented or absent.	The indicators are half partially or absent.	The indicators are somewhat presented or absent.	The indicators are not presented or absent.
Arms and hands (emphasized, big, shadowed, or omitted)				
Cheek/chin (double or emphasized)				
Genital area (disconnected upper body from lower, shadowed, or omitted)				
Physical abuse				
Nose (emphasized, big, or shadowed)				
Forehead (emphasized or shadowed)				
Eyebrows (emphasized or shadowed)				
Ears (double, emphasized, or shadowed)				
Standing hair				
Emotional abuse				
Slanted figure				
Tiny head				
Omission of hands				
Omission of legs and feet				
Monster and grotesque figure				
Disintegrated body				
Arm and leg asymmetry				
Omission of the neck				
Tiny figure				
Omission of eyes				
Shading of the face				
Feet being pressed together				

**Table 4:** Descriptive statistics of demographics, TEQ, and MSDQ of Thais vs. Indians.

Variables	Thais	Indians
Age M(SD)	14.8 (1.73)	15.2 (1.64)
Gender N (%)		
Female	135 (69.9%)	135 (69.9%)
Male	58 (30.1%)	58 (30.1%)
Traumatic events N (%)		
Car accident	7 (3.6%)	21 (10.9%)
Physical assault	33 (17.1%)	17 (8.8%)
Terror attack or war	0 (0%)	3 (1.6%)
Hospitalization due to illness	6 (3.1%)	43 (22.3%)
Sexual abuse	26 (13.5%)	26 (13.5%)
Loss of family member	5 (2.6%)	114 (59.1%)
Social exclusion	5 (2.6%)	15 (7.8%)
Verbal or emotional abuse	190 (98.4%)	0 (0%)
No event	2 (1%)	54 (28%)
Other events	0 (0%)	10 (5.2%
MSDQ Scales M(SD)		
Somatization	13.76 (3.93)	3.61 (3.12)
Depression	20.46 (5.63)	6.54 (5.46)
Dissociation	20.06 (5.23)	4.2 (4.12)
MSDQ Total	54.29 (12.76)	14.35 (10.4)

M = mean, SD = standard deviation.

**Table 5:** Descriptive statistics of Pictorial indicators by forms of abuse (the index is a summation of the scores indicating the level of obviousness for indicators in each form of abuse).

Variables	Thais	Indians
CPA Indicators M(SD)		
Nose	1.4 (0.89)	1.94 (1.003)
Forehead	1.05 (0.37)	1.61 (0.70)
Eyebrows	1.35 (0.84)	2.45 (1.07)
Ears	1.51 (1.06)	1.64 (0.84)
Hair	1.89 (1.26)	1.67 (0.89)
PA Index	7.22 (2.8)	9.33 (2.35)
CSA Indicators M(SD)		
Eyes	3.17 (1.34)	2.9 (1.04)
Arms and hands	1.78 (1.27)	3.1 (1.05)
Cheek or chin	2.04 (1.22)	2.2 (1.08)
Genital area	1.01 (0.14)	2.5 (1.31)
SA Index	8.02 (2.31)	10.6 (2.61)
CEA Indicators M(SD)		
Slanted figure	1.18 (0.57)	1.78 (0.98)
Tiny head	1.77 (1.13)	1.05 (0.29)
Omitted hands or arms	2.66 (1.43)	2.13 (1.41)
Omitted legs or feet	2.22 (1.46)	2.14 (1.42)
Feet pressed	1.47 (0.73)	1.07 (0.34)
Leg arm asymmetry	1.4 (0.95)	1.7 (0.89)
Omitted eyes	1.12 (0.6)	1.19 (0.54)
EA Index	11.81 (2.72)	11.06 (2.41)

**Table 6:** Group-wise comparison of categorical variables.

Variable	Thais N (%)	Indians N (%)	*p*-Value
Female	135 (69.9%)	135 (69.9%)	NS
Male	58 (30.1%)	58 (30.1%)	NS
Car accident	7 (3.6%)	21 (10.9%)	NS
Physical assault	33 (17.1%)	17 (8.8%)	<0.05
Terror attack or war	0 (0%)	3 (1.6%)	NS
Hospitalization due to illness	6 (3.1%)	43 (22.3%)	<0.001
Sexual abuse	26 (13.5%)	26 (13.5%)	NS
Loss of family member	5 (2.6%)	114 (59.1%)	<0.001
Social exclusion	5 (2.6%)	15 (7.8%)	<0.05
Verbal or emotional abuse	190 (98.4%)	0 (0%)	<0.001
No event	2 (1%)	54 (28%)	<0.001
Other events	0 (0%)	10 (5.2%)	0.001

NS = Nonsignificant.

**Table 7:** Group-wise comparison of age and MSDQ scores using Mann–Whitney U test (Ranks).

Variables	Nationality	N	Mean Rank	Sum of Ranks
Age	India	193	208.05	40,153.00
	Thailand	193	178.95	34,538.00
Somatization	India	193	101.66	19,621.00
	Thailand	193	285.34	55,070.00
Depression	India	193	105.94	20,446.00
	Thailand	193	281.06	54,245.00
Dissociation	India	193	99.85	19,270.50
	Thailand	193	287.15	55,420.50
MSDQ Total	India	193	99.41	19,187.00
	Thailand	193	287.59	55,504.00

**Table 8:** Group-wise comparison of age and MSDQ scores using the Mann–Whitney U test (Test Statistics).

Variables	Mann–Whitney U	Wilcoxon W	Z	*p*-Value
Age	15,817.000	34,538.000	−2.618	0.009
Somatization	900.000	19,621.000	−16.199	0.000 *
Depression	1725.000	20,446.000	−15.429	0.000 *
Dissociation	549.500	19,270.500	−16.509	0.000 *
MSDQ Total	466.000	19,187.000	−16.571	0.000 *

* *p* < 0.001.

**Table 9:** Group-wise comparison of ratings of pictorial indicators and index for forms of abuse using the Mann–Whitney U test (Ranks).

Variables	Nationality	N	Mean Rank	Sum of Ranks
Nose	India	193	225.54	43,530.00
	Thailand	193	161.46	31,161.00
Forehead	India	193	239.32	46,189.00
	Thailand	193	147.68	28,502.00
Eyebrows	India	193	248.96	48,049.50
	Thailand	193	138.04	26,641.50
Ears	India	193	209.56	40,444.50
	Thailand	193	177.44	34,246.50
Hair	India	193	192.19	37,093.50
	Thailand	193	194.81	37,597.50
PA Index	India	193	242.50	46,803.00
	Thailand	193	144.50	27,888.00
Eyes	India	193	173.32	33,450.50
	Thailand	193	213.68	41,240.50
Arms or hands	India	193	244.55	47,198.00
	Thailand	193	142.45	27,493.00
Cheek or chin	India	193	201.70	38,928.00
	Thailand	193	185.30	35,763.00
Genital area	India	193	255.13	49,240.50
	Thailand	193	131.87	25,450.50
SA Index	India	193	244.34	47,158.00
	Thailand	193	142.66	27,533.00
Slanted figure	India	193	229.52	44,297.50
	Thailand	193	157.48	30,393.50
Tiny head	India	193	158.89	30,666.00
	Thailand	193	228.11	44,025.00
Omitted hands or arms	India	193	173.62	33,508.50
	Thailand	193	213.38	41,182.50
Omitted legs or feet	India	193	191.05	36,873.50
	Thailand	193	195.95	37,817.50
Feet pressed	India	193	163.21	31,499.50
	Thailand	193	223.79	43,191.50
Leg or arm asymmetry	India	193	216.96	41,874.00
	Thailand	193	170.04	32,817.00
Omitted eyes	India	193	201.02	38,797.50
	Thailand	193	185.98	35,893.50
EA Index	India	193	179.62	34,667.00
	Thailand	193	207.38	40,024.00

**Table 10:** Group-wise comparison of ratings of pictorial indicators and index for forms of abuse using the Mann–Whitney U test (Test Statistics).

Variables	Mann–Whitney U	Wilcoxon W	Z	*p*-Value
Nose	12,440.000	31,161.000	−6.493	<0.001
Forehead	9781.000	28,502.000	−10.520	<0.001
Eyebrows	7920.500	26,641.500	−10.654	<0.001
Ears	15,525.500	34,246.500	−3.406	0.001
Hair	18,372.500	37,093.500	−0.258	NS
PA Index	9167.000	27,888.000	−8.725	<0.001
Eyes	14,729.500	33,450.500	−3.924	<0.001
Arms or hands	8772.000	27,493.000	−9.561	<0.001
Cheek or chin	17,042.000	35,763.000	−1.524	NS
Genital area	6729.500	25,450.500	−13.128	<0.001
SA Index	8812.000	27,533.000	−9.027	<0.001
Slanted figure	11,672.500	30,393.500	−7.904	<0.001
Tiny head	11,945.000	30,666.000	−8.656	<0.001
Omitted hands or arms	14,787.500	33,508.500	−3.915	<0.001
Omitted legs or feet	18,152.500	36,873.500	−0.500	NS
Feet pressed	12,778.500	31,499.500	−7.531	<0.001
Leg or arm asymmetry	14,096.000	32,817.000	−5.054	<0.001
Omitted eyes	17,172.500	35,893.500	−2.771	0.006
EA Index	15,946.000	34,667.000	−2.474	0.013

**Table 11:** Correlation between Age, MSDQ scores, CPA index, and pictorial indicators of CPA (N = 386).

Variables	Age	CPA Index	Nose	Forehead	Eyebrows	Ears	Hair
Age	1.000	0.273 **	0.168 **	0.175 **	0.203 **	0.149 **	0.087
Somatization	−0.071	−0.330 **	−0.299 **	−0.434 **	−0.488 **	−0.018	0.014
Depression	0.024	−0.329 **	−0.320 **	−0.403 **	−0.476 **	0.002	0.037
Dissociation	−0.106 *	−0.323 **	−0.328 **	−0.443 **	−0.479 **	−0.003	0.054
MSDQ Total	−0.042	−0.333 **	−0.323 **	−0.439 **	−0.498 **	0.004	0.040

* *p* < 0.05; ** *p* < 0.01.

**Table 12:** Correlation between Age, MSDQ scores, CSA index, and pictorial indicators of CSA (N = 386).

Variables	Age	CSA Index	Eyes	Arms and Hands	Cheek or Chin	Genital Area
Age	1.000	0.114 *	0.020	0.040	0.156 **	0.112 *
Somatization	−0.071	−0.348 **	0.059	−0.340 **	−0.022	−0.549 **
Depression	0.024	−0.319 **	0.085	−0.292 **	−0.031	−0.509 **
Dissociation	−0.106 *	−0.316 **	0.094	−0.331 **	−0.012	−0.508 **
MSDQ Total	−0.042	−0.337 **	0.078	−0.324 **	−0.025	−0.538 **

* *p* < 0.05; ** *p* < 0.01.

**Table 13:** Correlation between Age, MSDQ scores, CEA index, and pictorial indicators of CEA (N = 386).

Variables	Age	Somatization	Depression	Dissociation	MSDQ Total
Age	1	−0.071	0.024	−0.106 *	−0.042
CEA	0.047	0.105 *	0.129 *	0.141 **	0.126 *
Slanted figure	−0.163 *	−0.122	−0.111	−0.034	−0.126
Tiny head	0.019	−0.115	−0.087	0.000	−0.086
Omitted hands	0.030	−0.030	0.006	0.078	0.021
Omitted legs	0.075	−0.072	0.019	0.033	0.007
Arm or leg Asymmetry	−0.225 **	−0.011	−0.146 *	−0.078	−0.114
Omitted neck	0.037	0.046	−0.028	−0.076	0.002
Tiny figure	−0.055	−0.144 *	−0.064	−0.124	−0.109
Omitted eyes	−0.084	−0.033	0.078	−0.036	0.009
Shading of face	−0.096	0.028	0.014	0.183 *	0.056
Feet pressed	0.017	−0.113	−0.001	−0.004	−0.031

* *p* < 0.05; ** *p* < 0.01.

## Data Availability

The original contributions presented in the study are included in the article, further inquiries can be directed to the corresponding author.
